# Dual Anti-OX40/IL-2 Therapy Augments Tumor Immunotherapy via IL-2R-Mediated Regulation of OX40 Expression

**DOI:** 10.1371/journal.pone.0034467

**Published:** 2012-04-04

**Authors:** William L. Redmond, Todd Triplett, Kevin Floyd, Andrew D. Weinberg

**Affiliations:** 1 Robert W. Franz Cancer Research Center, Earle A. Chiles Research Institute, Providence Portland Medical Center, Portland, Oregon, United States of America; 2 Department of Molecular Microbiology and Immunology, Oregon Health & Science University, Portland, Oregon, United States of America; University of Montreal, Canada

## Abstract

The provision of T cell co-stimulation via members of the TNFR super-family, including OX40 (CD134) and 4-1BB (CD137), provides critical signals that promote T cell survival and differentiation. Recent studies have demonstrated that ligation of OX40 can augment T cell-mediated anti-tumor immunity in pre-clinical models and more importantly, OX40 agonists are under clinical development for cancer immunotherapy. OX40 is of particular interest as a therapeutic target as it is not expressed on naïve T cells but rather, is transiently up-regulated following TCR stimulation. Although TCR engagement is necessary for inducing OX40 expression, the downstream signals that regulate OX40 itself remain unclear. In this study, we demonstrate that OX40 expression is regulated through a TCR and common gamma chain cytokine-dependent signaling cascade that requires JAK3-mediated activation of the downstream transcription factors STAT3 and STAT5. Furthermore, combined treatment with an agonist anti-OX40 mAb and IL-2 augmented tumor immunotherapy against multiple tumor types. Dual therapy was also able to restore the function of anergic tumor-reactive CD8 T cells in mice with long-term well-established (>5 wks) tumors, leading to increased survival of the tumor-bearing hosts. Together, these data reveal the ability of TCR/common gamma chain cytokine signaling to regulate OX40 expression and demonstrate a novel means of augmenting cancer immunotherapy by providing dual anti-OX40/common gamma chain cytokine-directed therapy.

## Introduction

In addition to B7-CD28 co-stimulation, members of the tumor necrosis factor receptor (TNFR) super-family, including OX40 (CD134), 4-1BB (CD137), and CD27 can augment T cell responses [1], [2]. Specifically, OX40 ligation can augment T cell differentiation, cytokine production, the generation of memory T cells, and it can affect the generation and function of regulatory CD4 T cells [3], [4]. Pre-clinical studies have shown that ligation of OX40 via agonist anti-OX40 mAb or OX40L-Ig fusion proteins can drive robust T cell-mediated anti-tumor immunity [1], [3]. Based upon these data, a phase 1 clinical trial was performed with an agonist anti-human OX40 mAb for the treatment of patients with cancer. Additional studies are underway to explore the efficacy of combining OX40-targeted therapy with other modalities such as chemotherapy or radiation therapy.

One of the major advantages of targeting OX40 is the restricted nature of OX40 expression. OX40 is not expressed on naïve T cells and is transiently up-regulated 24–120 hours following TCR ligation [5], [6]. TCR stimulation drives OX40 expression in a dose-dependent manner as high-doses of cognate Ag induced maximal OX40 expression, while weak TCR stimulation led to poor induction of OX40 [5], [7]. Although TCR stimulation is necessary to up-regulate OX40, additional signals are required for inducing optimal OX40 expression. For example, CD28 signaling can contribute to OX40-mediated co-stimulation [8], [9], although CD28 itself is not required for the generation of OX40-dependent responses [10], [11]. Since CD28 ligation leads to increased IL-2Ralpha (CD25) expression and IL-2 production [12], it is unclear whether CD28-B7 signaling contributes to OX40-mediated co-stimulation directly or through an IL-2-dependent mechanism. IL-2R signaling can also modulate OX40-dependent co-stimulation as OX40 ligation drives increased IL-2 production and CD25 expression on T cells [13], [14], [15], while CD25-deficient T cells exhibited defective differentiation following OX40 engagement [10], [16]. However, these studies did not address directly whether IL-2R signaling affects OX40 expression.

IL-2/IL-2R signaling occurs via the trimeric IL-2 receptor which consists of the IL-2Ralpha (CD25), IL-2/IL-15Rbeta (CD122), and common gamma (gc; CD132) chains [17]. IL-2R signaling is initiated by phosphorylation of JAK3 and JAK1, which are constitutively associated with the gc and IL-2Rbeta chains, respectively. Activation of these kinases leads to the activation of PI3K/AKT, MAPK/ERK, and the STAT family of transcription factors [18]. Other IL-2 family members also utilize the gc subunit including IL-4, IL-7, IL-9, IL-15, and IL-21. Importantly, whether IL-2R and/or common gc cytokine signaling regulates OX40 expression remains controversial. While IL-2 and IL-4 can up-regulate OX40 expression, others have shown that IL-2R signaling was dispensable for inducing OX40 [7], [10], [19].

In this study, we demonstrate that OX40 expression is driven via a dual TCR/common gc cytokine-dependent signaling pathway that was dependent upon activation of JAK3 and the transcription factors STAT3 and STAT5. Furthermore, combined targeting of OX40 in conjunction with IL-2 therapy enhanced tumor regression in several different pre-clinical tumor models and was able to restore the function of anergic tumor-reactive CD8 T cells in mice with long-term well-established tumors, leading to enhanced survival of the tumor-bearing mice. Together, these data provide insight into the regulation of the OX40 co-stimulatory receptor by TCR/gc cytokine signaling and suggest that combined anti-OX40/gc cytokine-directed therapy can provide a novel strategy to boost tumor immunotherapy and revive the function of tumor-reactive CD8 T cells for the treatment of patients with cancer.

## Methods

### Ethics Statement

The Providence Health System Institutional Review Board approved the study and all blood donors gave their informed written consent. All mice were maintained under specific pathogen-free conditions in the Providence Portland Medical Center animal facility and experimental procedures were performed according to the National Institutes of Health Guide for the Care and Use of Laboratory Animals under protocol #39 approved by the PPMC Institutional Animal Care and Use Committee.

### Mice

Wild-type and CD25^+/−^ C57BL/6 mice were purchased from Jackson Labs (Bar Harbor, ME). C57BL/6 OX40-Cre mice were provided by Dr. Killeen (UCSF, San Francisco, CA) and were crossed to mice carrying the *Rosa26*-*loxP*-STOP-*loxP*-YFP allele [20]. Splenocytes from STAT3^−/−^ OT-I TCR Tg mice were provided by Dr. Yu (Beckman Research Institute at City of Hope, Duarte, CA). OT-I Thy1.1 TCR Tg, POET-1 Tg, OX40^−/−^ OT-I TCR Tg, and STAT5a/b^+/−^ mice were bred in our facility. All mice were maintained under specific pathogen-free conditions in the Providence Portland Medical Center animal facility. Experimental procedures were performed according to the National Institutes of Health Guide for the Care and Use of Laboratory Animals.

### Lymphocyte isolation and analysis

Lymph nodes were harvested and processed to obtain single cell suspensions. ACK lysing buffer (Lonza, Walkersville, MD) was added for 5 min at RT to lyse red blood cells. Cells were then rinsed with RPMI 1640 medium (Lonza) containing 10% FBS (10% cRPMI) (Lonza) supplemented with 1 M HEPES, non-essential amino acids, sodium pyruvate (all from Lonza), and pen-strep glutamine (Invitrogen). Murine peripheral blood lymphocytes were collected via the tail vein into tubes containing 50 mcl heparin (Hospira, Lake Forest, IL). One ml of flow cytometry wash buffer (0.5% FBS, 0.5 mM EDTA, and 0.02% NaN_3_ in PBS) was added, cells were mixed, and then 700 mcl of Ficoll-Paque (GE Healthcare, Piscataway, NJ) was added prior to centrifugation. Lymphocytes were collected from the interface and then washed with flow cytometry buffer prior to staining. Human PBMC from healthy donors were isolated by centrifugation of heparinized blood over Ficoll-Paque PLUS (GE Healthcare). The Providence Health System Institutional Review Board approved the study and all blood donors gave their informed consent. Fresh human PBMC were enriched for CD4 and CD8 T cells by negative selection using a CD4 or CD8 T cell negative isolation kit (Miltenyi Biotec), suspended in 10% cRPMI (5×10^5^ cells/ml), and stimulated with 1 µg/ml plate bound anti-CD3 (clone OKT-3) in 96-well plates with or without 5,000 U/ml of rhIL-2 (Proleukin). After 48 hours, cells were washed, re-suspended, and then plated in 96-well plates +/− 5,000 IU/ml of rhIL-2.

### Flow cytometry

Murine cells were stained for 30 min at 4°C with: Ki-67 FITC, Thy1.1 PE-Cy7, Thy1.1 eFluor 450, OX40 PE, granzyme B PE, CD3 eFluor 710, CD8 eFluor 605, CD8 PE-Cy7, KLRG-1 APC, CD25 eFluor 488, CD25 Alexa Fluor 700, Viability Dye eFluor 780, or CD4 V500. Human cells were incubated with CD3 APC-H7, CD4 PerCP-Cy5.5, CD8 PE-Cy7, APC CD25 and OX40 PE. All antibodies were obtained from eBioscience (San Diego, CA), BD Biosciences (San Jose, CA), BioLegend (San Diego, CA), Miltenyi Biotec (Bergisch Gladbach, Germany), or Invitrogen. For intracellular staining, cells were fixed and permeabilized with the Foxp3 Staining Buffer Set (eBioscience) according to the manufacturer's instructions. Cells were analyzed with an LSR II flow cytometer using FACSDiva software (BD Biosciences).

### Western blotting

Whole cell lysates were prepared using RIPA lysis buffer (Bio-Rad, Hercules, CA) containing HALT protease inhibitor cocktail (Thermo Fisher Scientific, Rockford, IL) for 30 min at 4 C. Lysates were centrifuged at 14,000 g/4°C, supernatants were collected, protein concentration was determined by Bradford assay kit (ISC BioExpress, Kaysville, UT) and 50 mcg aliquots were stored at −80°C. Lysates were boiled at 100°C for 5 min in Laemmli buffer (Invitrogen) containing 2-ME, resolved by SDS-PAGE on 12% pre-cast gels (Bio-Rad), and then transferred to nitrocellulose membranes (Invitrogen). Non-specific binding was reduced by blocking with a 1∶1 mixture of Odyssey Blocking buffer (Li-Cor, Lincoln, NE) and 1X PBS or 5% non-fat dry milk in 1X PBS for 1 hour at RT. Blots were incubated with Abs against pJAK1, pJAK2, pSTAT1, pSTAT3, pSTAT5, pSTAT6, JAK1, JAK2, STAT1, STAT3, STAT4, STAT5, STAT6 (all from Cell Signaling, Danvers, MA), pJAK3, JAK3 (Santa Cruz Biotechnology, Santa Cruz, CA), pSTAT4 (Invitrogen), GAPDH (Sigma), or beta-actin (Li-Cor) in Odyssey (Li-Cor) blocking buffer overnight at 4°C. Blots were washed with PBS-Tween (1X PBS+0.2% Tween-20) and then incubated with IRDye 800CW goat anti-rabbit IgG, IRDye 680LT goat anti-mouse IgG, or IRDye 680LT donkey anti-Goat IgG (Li-Cor) for 60 min at RT. Blots were washed with PBS-Tween and then rinsed with 1X PBS prior to image acquisition (Li-Cor Odyssey).

### Cytokines and inhibitors

Recombinant murine IL-2, IL-4, IL-7, IL-9, or IL-21 were purchased from eBioscience or Peprotech (Rocky Hill, NJ). Recombinant human IL-15 was provided by the National Cancer Institute's Biological Resources Branch and anti-mIL-2 mAb (clone S4B6) was obtained from Bio-X-Cell. IL-2/anti-IL-2 mAb complexes (IL-2c) were generated by mixing 2.5 mcg IL-2 with 7 mcg anti-IL-2 mAb for 20 min at 37°C and then mice received daily injections of IL-2c in 200 mcl PBS (i.p.). Where indicated, T cells were treated in vitro with a JAK3 inhibitor (100 ng/ml; PF-956980; Pfizer).

### *In vivo* cytolytic assay

Target cells, comprised of syngeneic splenocytes, were labeled with 5 mcM CFSE (CFSE^high^) or 0.5 mcM CFSE (CFSE^low^) in 1X PBS for 10 minutes at 37°C and then washed twice with 10% cRPMI. Next, CFSE^low^ and CFSE^high^ cells were pulsed with 5 mcg/ml control (HA) or cognate (OVA) peptide, respectively, for 1 h at 37°C. Cells were washed with 10% cRPMI and then a 1∶1 mixture of CFSE^low^/CFSE^high^ target cells (5×10^6^/each) were injected i.v. in 1X PBS into recipient mice. Four hours later, splenocytes were harvested and analyzed for detection and quantification of CFSE-labeled cells by flow cytometry.

### Treg functional assay

MCA-205 tumors were implanted into wild-type C57BL/6 mice and then 10 days later, mice received 250 mcg anti-OX40 or control rat Ig (d10, 14; i.p.) +/− IL-2c (d10-13; i.p.). Seven days later (d21 post-tumor implantation), spleens were harvested, RBC lysed, and CD4^+^CD25^+^ regulatory T cells (CD8^−^/MHC II^−^/B220^−^) were isolated by cell sorting (>99% purity). Treg were seeded in triplicate at 5×10^4^ cells/well in 96-well round-bottom plates. Naïve responder (Teff) CD8 cells were prepared from the spleens of wild-type mice using the Dynal CD8 T cell negative selection kit (Invitrogen), CFSE-labeled, and 5×10^4^ cells/well were added to triplicate wells containing media (positive control) or Treg cells. 2×10^5^ irradiated (4,000 rads) T-cell depleted (Dynal beads, Invitrogen) accessory cells were prepared, treated with 1 mcg/ml anti-CD3 and added to all wells. Cells were harvested 96 hours later, stained for CD8, and the extent of CFSE dilution in the CD8 responder cells was determined by flow cytometry.

### Adoptive transfer and activation of OT-I T cells in vivo

Single cell suspensions were prepared from the spleens of OT-I Thy1.1 TCR Tg mice. OT-I T cells were purified by negative selection using the Dynal mouse CD8 cell isolation kit (Invitrogen, Carlsbad, CA) and were injected i.v. in 200 mcl of PBS into recipient mice. Where indicated, recipient mice received 500 mcg of soluble ovalbumin (Sigma, St. Louis, MO), 50 mcg anti-OX40 (clone OX86) or control rat IgG Ab (Sigma), and/or 10 mcg bacterial lipopolysaccharide (LPS) (Sigma) s.c. Mice received an additional dose (50 mcg) of anti-OX40 or control Ab one day later. For cell depletion, tumor-bearing mice were treated with 200 mcg (i.p.) anti-CD4 (clone GK1.5; Bio X Cell, West Lebanon, NH) and/or anti-CD8 (clone 53-6.72; Bio X Cell).

### T cell activation *in vitro*

Single cell suspensions were prepared from the lymph nodes and spleens of wild-type, CD25^−/−^, STAT3^−/−^, or STAT5^−/−^ mice and then CD4 or CD8 T cells were purified using the Dynal mouse CD4 or CD8 T cell negative isolation kit (Invitrogen). 3×10^5^ cells per well were seeded into 96-well plates containing plate-bound anti-CD3 (1 mcg/ml; clone 145-2C11) and anti-CD28 (5 mcg/ml; clone 37.51). For Ag-specific CD8 T cell activation, purified naïve wild-type or OX40^−/−^ OT-I T cells (2×10^5^/well) were stimulated with OVA peptide (SIINFEKL)-pulsed irradiated (20,000 rads) DC2.4 cells (2×10^3^/well) in 96-well plates. Alternatively, purified naïve wild-type OT-I, STAT3^−/−^ OT-I, or OX40^−/−^ OT-I T cells (1×10^6^/well) were stimulated with wild-type cognate (SIINFEKL) or altered peptide ligand (SIITFEKL) OVA peptide-pulsed irradiated (2,000 rads) syngeneic splenocytes (6×10^6^/well) in 24-well plates. Forty-eight hours later, activated OT-I T cells were harvested and live cells were enriched over a Ficoll-paque gradient prior to re-seeding in 10% cRPMI (5×10^5^ cells/ml) +/− cytokines.

### Tumor challenge and anergy induction

1×10^6^ MCA-205 sarcoma tumor cells were implanted into C57BL/6 mice (s.c.). MCA-205 cells were kindly provided by Dr. Suyu Shu (Cleveland Clinic, Cleveland, OH) [21]. TRAMP-C1-mOVA (TC1-OVA) cells were generated as previously described [16]. In some experiments, 2.5×10^6^ TC1-OVA cells were injected into male POET Tg mice (s.c.). When tumors reached ∼50 mm^2^ (20 days post-tumor inoculation), mice received either 5×10^5^ wild-type OT-I Thy1.1 T cells. Seventeen days after CD8 T cell adoptive transfer, anergic donor cells in tumor-bearing mice were re-challenged with soluble OVA, anti-OX40 or control Ab, and LPS (s.c.) as described above. Tumor growth (area) was assessed every 2–3 days with micro-calipers and mice were sacrificed when tumors reached >150 mm^2^.

### Statistical analysis

Statistical significance was determined by unpaired Student's t-test (for comparison between 2 groups), one-way ANOVA (for comparison among >2 groups), or Kaplan-Meier survival (for tumor survival studies) using GraphPad InStat or Prism software (GraphPad, San Diego, CA); a P value of <0.05 was considered significant.

## Results

### Optimal OX40 expression is regulated by the strength of TCR stimulation and IL-2Ralpha (CD25)

**Figure 1:**
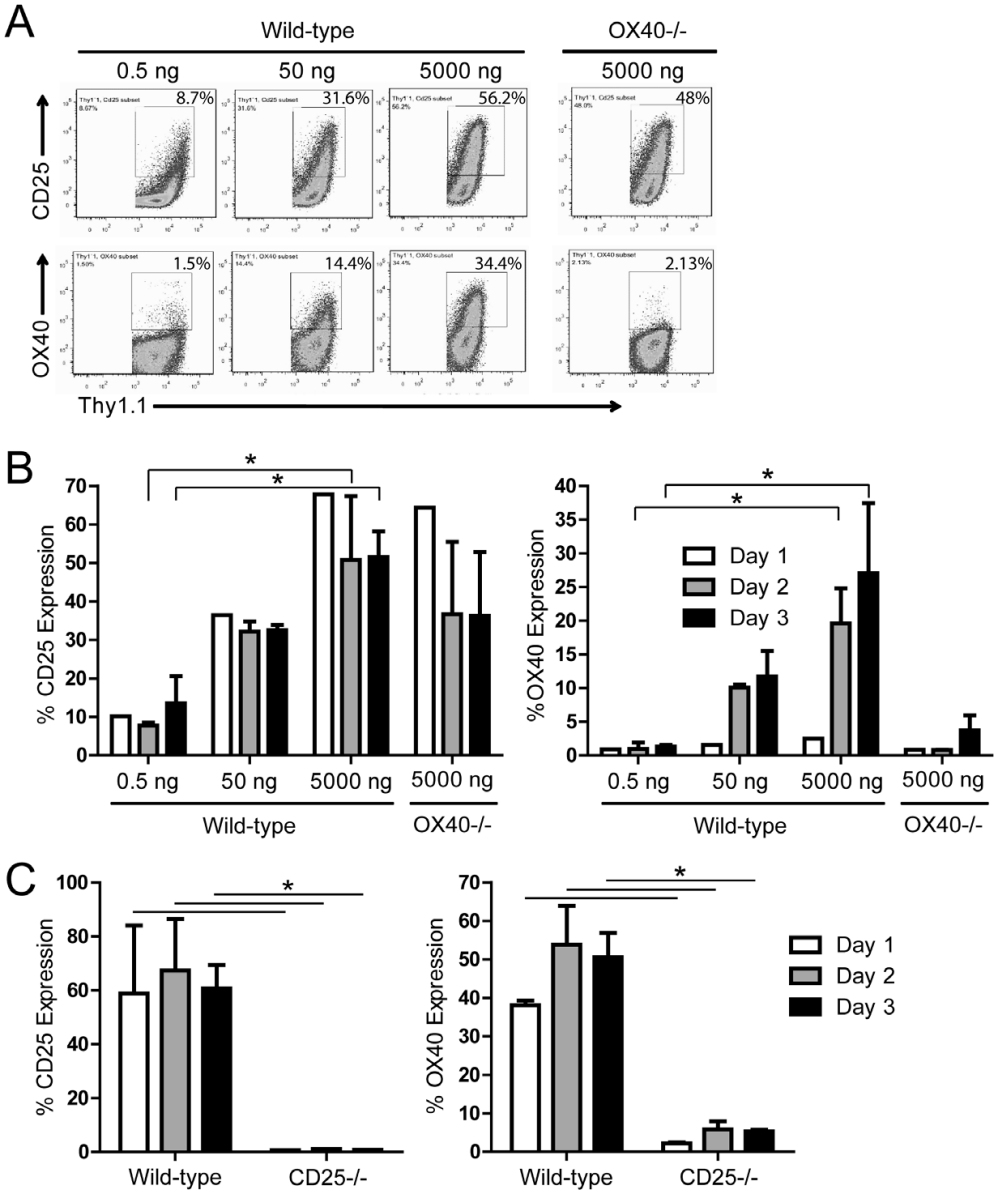
OX40 is regulated by TCR stimulation and IL-2Ralpha (CD25) expression. (**A, B**) Naïve wild-type or OX40^−/−^ OT-I T cells (2×10^5^/ml) were stimulated with peptide-pulsed APCs (2×10^3^/ml). **A**) Three days later, OT-I T cells were harvested and the extent of CD25 and OX40 expression were determined. **B**) Kinetics of CD25 and OX40 expression following TCR stimulation were determined at the indicated time points by flow cytometry. (**C**) Naïve polyclonal wild-type or CD25^−/−^ CD8 T cells (3×10^5^/well) were CFSE-labeled and then stimulated with anti-CD3 and anti-CD28 (1 and 5 mcg/ml, respectively). One to three days later, CD8 T cells were harvested and the extent of CD25 and OX40 expression were determined. **B, C**) Bar graphs depict the mean+/−SD (n = 2–3/group). Data are representative of one out of two to three independent experiments. *P<0.05.

To assess the extent to which the strength of TCR stimulation affects OX40 expression, we examined the kinetics of OX40 up-regulation following CD8 T cell activation. Purified naïve wild-type or OX40-deficient OT-I CD8 T cells were stimulated with increasing doses of cognate peptide. One to three days later, activated OT-I T cells were harvested and the expression of OX40 and CD25 were determined. CD25 was rapidly up-regulated and reached maximal expression within 24 hrs after TCR stimulation at the highest dose of Ag (5000 ng/ml) whether or not OX40 was expressed (Fig. 1A, 1B). Maximal OX40 expression was similarly induced in a dose-dependent manner with peak OX40 expression observed 3 days post-stimulation (Fig. 1A, 1B).

Next, we determined the effects of IL-2 on OX40 expression on T cells. Polyclonal wild-type or CD25^−/−^ CD8 T cells were CFSE-labeled and then stimulated with anti-CD3 and anti-CD28 Abs. One to three days later the cells were harvested and the extent of CD25 and OX40 expression was assessed. CD25 and OX40 were both induced on wild-type T cells (Fig. 1C), while CD25^−/−^ CD8 T cells expressed little or no OX40 following TCR stimulation (Fig. 1C), demonstrating that TCR stimulation alone was not sufficient to drive robust expression of OX40. The lack of OX40 expression was not due to differences in proliferation as wild-type and CD25^−/−^ CD8 T cells divided to a similar extent (based upon CFSE dilution) (Figure S1). Similar results were obtained following stimulation of murine polyclonal CD25^−/−^ CD4 T cells (data not shown), demonstrating that expression of the high-affinity IL-2R complex is required for optimal induction of OX40 on T cells.

### Exogenous IL-2 up-regulates OX40 on activated murine and human T cells

**Figure 2:**
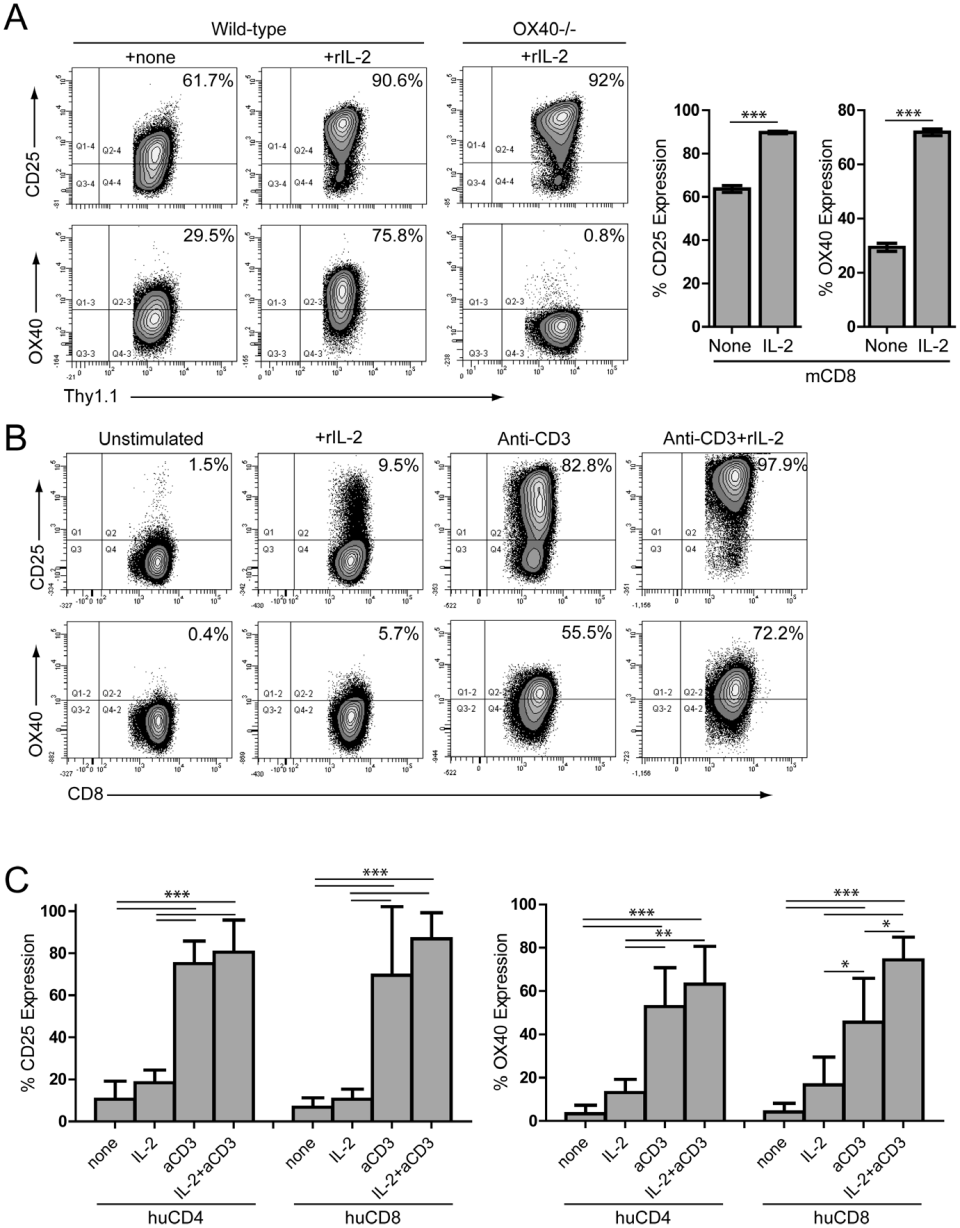
OX40 is regulated on murine and human T cells by TCR stimulation and IL-2. **A**) Purified naïve wild-type or OX40^−/−^ OT-I T cells (1×10^6^/ml) were stimulated with peptide-pulsed APCs (6×10^6^/ml). Two days later, OT-I T cells were harvested and re-cultured (5×10^5^ cells/ml) +/− rmIL-2 (100 ng/ml). Twenty-four hours later, cells were harvested and the extent of CD25 and OX40 expression were determined. Bar graphs depict the mean+/−SEM (n = 6/group). **B, C**) Human CD8 or **C**) CD4 T cells collected from PBMC were stimulated with media, rhIL-2 (5,000 IU/ml, equivalent to 300 ng/ml), and/or 1 mcg/ml anti-CD3 mAb (OKT-3). Forty-eight hours later, cells were harvested, washed, and stimulated with media or rhIL-2 (5,000 IU/ml). Twenty-four hours later, the extent of CD25 and OX40 expression were measured. **C**) Bar graphs depict the mean+/−SD (n = 3–5/group). Data are pooled from five independent experiments. *P<0.05; **P<0.01; ***P<0.001.

To determine whether the addition of exogenous rIL-2 was sufficient to up-regulate OX40 on activated T cells, naïve wild-type or OX40^−/−^ OT-I CD8 T cells were stimulated with peptide-pulsed APCs for 2 days and then re-cultured with media (control) or rIL-2 and the extent of CD25 and OX40 expression was determined. Stimulation with exogenous rIL-2 led to a statistically significant increase in both CD25 and OX40 expression compared to media alone (Fig. 2A), demonstrating that IL-2 signaling was sufficient to drive up-regulation of these molecules. Next, we examined whether TCR stimulation plus exogenous rIL-2 similarly regulated OX40 expression on human T cells. Freshly isolated purified human CD8 or CD4 T cells were stimulated with anti-CD3+/− rhIL-2 and the expression of CD25 and OX40 were determined. CD25 and OX40 were both modestly induced following exposure to IL-2 (Figs. 2B, 2C). Although stimulation with anti-CD3 alone led to significantly increased CD25 expression, combined rhIL-2 and TCR stimulation trended towards increased OX40 expression on human CD4 T cells (Fig. 2C) and a statistically significant increase in OX40 on human CD8 T cells (Figs. 2B, 2C). Together, these data demonstrate that the combination of TCR/IL-2R stimulation induced optimal OX40 expression on murine and human T cells.

### OX40 expression is regulated by JAK3, STAT3, and STAT5

**Figure 3:**
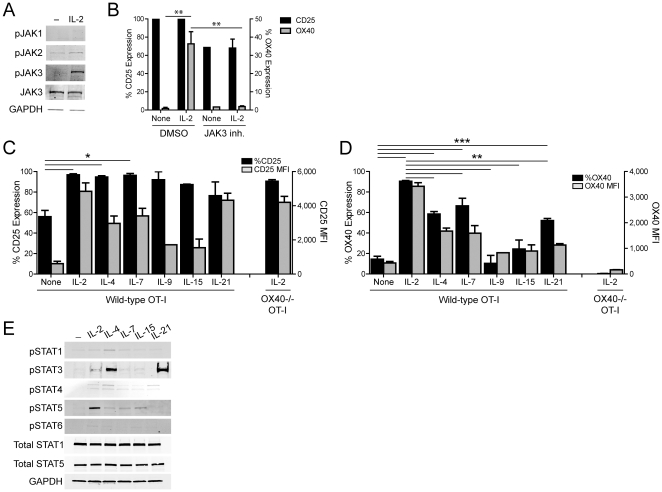
Common gc cytokines regulate OX40 via JAK/STAT signaling. **A**) Naïve WT OT-I T cells were stimulated with peptide-pulsed APCs (as in Fig. 3A). **A**) Two days later, OT-I T cells were harvested and re-cultured (5×10^5^ cells/ml) with media or rmIL-2 (100 ng/ml) and the expression of the indicated proteins was assessed by Western blot. **B**) WT OT-I T cells were stimulated (as in (**A**)) +/− a JAK3 inhibitor (PF-956980; 100 ng/ml). Twenty-four hours later, cells were harvested and the extent of CD25 and OX40 expression was determined. **C, D**) WT or OX40^−/−^ OT-I cells were stimulated for 2 days, harvested, and then re-stimulated with media alone, rmIL-2, rmIL-4, rmIL-7, rmIL-9, rmIL-15, or rmIL-21 (100 ng/ml). Twenty-four hours later, cells were harvested and the extent of **C**) CD25 and **D**) OX40 expression (% positive and MFI) were determined. **E**) WT OT-I T cells were activated and then re-stimulated with the indicated common gc cytokines and protein expression was assessed by Western blot. **B–D**) Bar graphs depict the mean+/−SD from **B**) n = 2–3/group or **C, D**) n = 3–8/group. Data are representative of one out of two to ten independent experiments. *P<0.05; ** P<0.01; *** P<0.001.

The tyrosine kinase JAK3 binds to the common gc subunit and its phosphorylation is a critical factor in the proximal downstream signaling following stimulation with gc cytokines [22], [23]. To examine whether JAK3 was required to induce OX40 expression, we first examined the expression of JAK proteins in CD8 T cells stimulated *in vitro*. We used Ag-specific CD8 T cells (as in Fig. 3A) for these studies in order to control more precisely the extent and duration of TCR stimulation. Naïve wild-type or OX40^−/−^ OT-I T cells were activated for two days and then stimulated with media or rIL-2. Twenty-four hours later, the cells were harvested and protein expression was assessed. Stimulation with rIL-2 led to increased phosphorylation of JAK3, but did not affect JAK1 or JAK2 phosphorylation (Fig. 3A). Furthermore, treatment with a JAK3 inhibitor (PF-956980) [24] abrogated the IL-2-mediated induction of OX40 on activated CD8 T cells compared to control-treated cells (DMSO) (Fig. 3B).

The gc subunit is constitutively expressed and shared among the following cytokines: IL-2, IL-4, IL-7, IL-9, IL-15, and IL-21. Despite sharing the common gc subunit, the majority of IL-2 family cytokines signal through a complex consisting of a unique alpha chain paired with the shared gc, which leads to distinct downstream effects on T cell survival and differentiation [17], [22]. To determine how the different gc cytokines affected OX40 expression, OT-I T cells were cultured in the presence of IL-2, IL-4, IL-7, IL-9, IL-15, or IL-21 (as in Fig. 3A) and CD25 and OX40 expression were measured. While all the gc cytokines tested were able to induce increased expression of CD25 (Fig. 3C), IL-2 stimulation was uniquely able to promote maximal OX40 expression (Fig. 3D, % OX40^+^). In contrast, stimulation with IL-4, IL-7, or IL-21 led to a modest up-regulation of OX40 (Fig. 3D; %OX40^+^), while IL-9 and IL-15 did not affect OX40 (Fig. 3D).

JAK3 and gc cytokines promote T cell activation and survival through three major pathways, PI3K/AKT, MAPK/ERK, and the activation of STAT transcription factors [25]. No change in the IL-2-mediated induction of OX40 was observed following activation in the presence of PI3K or AKT inhibitors (data not shown). Similarly, wild-type and ERK2^−/−^ CD8 T cells expressed similar amounts of OX40 (data not shown), demonstrating that OX40 was induced independently of PI3K/AKT or ERK. These data led us to investigate the role of STAT signaling in driving OX40 expression. As seen in Fig. 3E, IL-2 stimulation led to a robust increase in STAT5 phosphorylation, while IL-4, IL-7, and IL-15 caused lower levels of STAT5 phosphorylation (Fig. 3E). Conversely, IL-21 and IL-4 induced high levels of STAT3 phosphorylation, while IL-2 weakly induced STAT3 phosphorylation. Further analysis revealed no differential expression and only low levels of STAT1, STAT4, and STAT6 phosphorylation (Fig. 3E).

**Figure 4:**
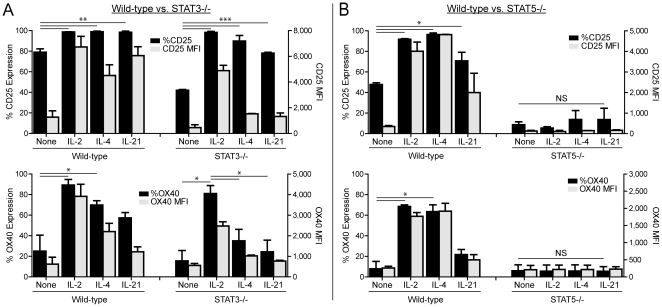
STAT3 and STAT5 are required for optimal up-regulation of OX40 following stimulation with common gc cytokines. **A**) WT or STAT3^−/−^ OT-I T cells were stimulated for 2 days, harvested, and then re-cultured with media alone, rmIL-2, rmIL-4, or rmIL-21 (100 ng/ml); 24 hours later cells were harvested and the extent of CD25 and OX40 expression (% positive and MFI) were measured. **B**) Polyclonal endogenous WT or STAT5^−/−^ CD8 T cells were stimulated for 2 days with 2 mcg/ml anti-CD3 mAb, harvested, and then re-cultured with media alone, rmIL-2, rmIL-4, or rmIL-21 (100 ng/ml) and then 24 hours later, cells were harvested and the extent of CD25 and OX40 expression (% positive and MFI) were determined. **A, B**) Bar graphs depict the mean+/−SD (n = 2–3/group). Data are representative of one out of two independent experiments. *P<0.05; ** P<0.01; *** P<0.001; NS = no statistically significant difference.

To address directly the contribution of STAT3 and STAT5 to the regulation of OX40, wild-type, STAT3^−/−^, or STAT5^−/−^ CD8 T cells were activated for 2 days and then stimulated with media, IL-2, IL-4, or IL-21. We focused on these cytokines because they up-regulated CD25 and OX40 and induced strong phosphorylation of STAT3 and/or STAT5. Twenty-four hours later, the cells were harvested and the extent of CD25 and OX40 expression (% positive and MFI) were determined. Both wild-type and STAT3^−/−^ CD8 T cells up-regulated CD25 following stimulation with gc cytokines, although STAT3^−/−^ CD8 T cells exhibited reduced expression (% positive and MFI) compared to wild-type cells, particularly following stimulation with IL-4 or IL-21 (Fig. 4A). However, only IL-2 and not IL-4 or IL-21 was sufficient to induce significant up-regulation of OX40 on STAT3^−/−^ CD8 T cells (Fig. 4A; % OX40^+^).

Conversely, STAT5-deficient CD8 T cells were unable to induce CD25 or OX40 expression following stimulation with IL-2, IL-4, or IL-21 indicating an essential role for STAT5 in driving gc cytokine-mediated up-regulation of CD25 and OX40 (Fig. 4B). It should be noted that similar results were obtained using either TCR Tg OT-I T cells (Fig. 4A) stimulated with cognate peptide or endogenous polyclonal CD8 T cells activated with anti-CD3 (Fig. 4B and data not shown). Together, these studies demonstrated that gc cytokines regulate OX40 via unique mechanisms as IL-2 drove OX40 expression in a primarily STAT3-independent and STAT5-dependent manner, while IL-4 and IL-21 induced OX40 via a dual STAT3/STAT5-dependent mechanism.

### Combined anti-OX40 mAb/IL-2 therapy boosts anti-tumor immunity

**Figure 5:**
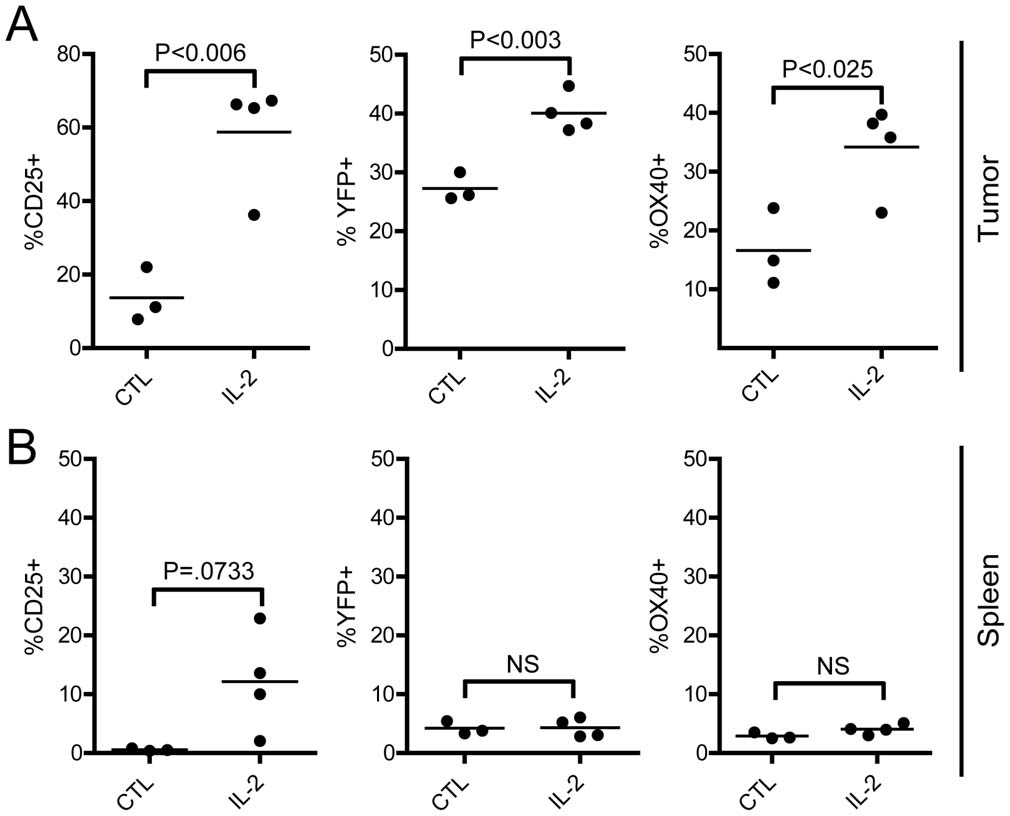
IL-2 treatment enhanced OX40 expression on CD8 T cells in tumor-bearing hosts. **A, B**) C57BL/6 OX40-cre x ROSA-YFP reporter mice received 1×10^6^ MCA-205 sarcoma tumor cells (day 0) and two weeks later, the tumor-bearing mice were treated with IL-2 cytokine/mAb complexes (day 14, 15; i.p.). Twenty four hours later (day 16 post-tumor inoculation) the extent of CD25, YFP (OX40 reporter), and OX40 expression on CD8 T cells isolated from the **A**) tumor and **B**) spleen were assessed. Graphs depict the results obtained from 3–4 individual animals from 1 out of 2 independent experiments.

Based upon the ability of IL-2 to strongly induce OX40 *in vitro* (Fig. 2), we sought to evaluate whether the provision of IL-2 in conjunction with anti-OX40 mAb therapy would augment anti-tumor immunity *in vivo*. First, we confirmed whether IL-2 stimulation was capable of up-regulating OX40 *in vivo* on CD8 T cells in tumor-bearing mice. IL-2 was provided via cytokine/mAb complexes (IL-2c) in order to minimize the deleterious side-effects associated with systemic rIL-2 therapy [26], [27]. Since OX40 expression is often difficult to detect on CD8 T cells stimulated *in vivo*, we utilized the recently described OX40-cre x ROSA-YFP reporter mice to identify OX40-expressing CD8 T cells [28]. These data revealed that IL-2 treatment significantly enhanced CD25 and OX40 expression on CD8 T cells localized in the tumor (Fig. 5A), while no significant differences were detected on CD8 T cells in the spleen (Fig. 5B).

**Figure 6:**
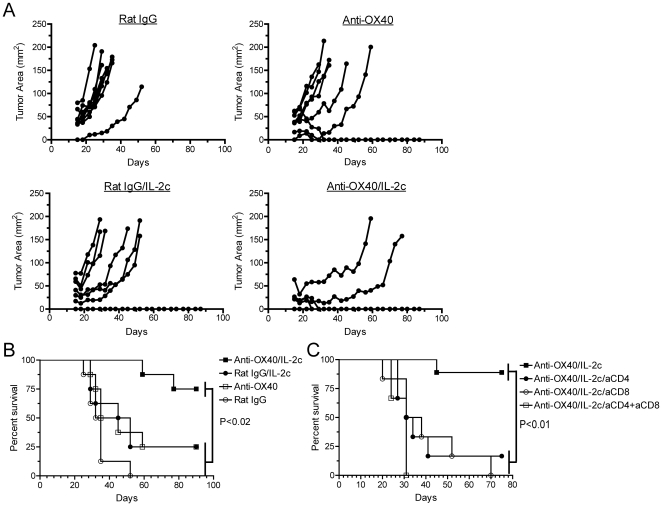
Combined anti-OX40/IL-2c therapy boosts anti-tumor immunity through a T cell-dependent mechanism. **A, B**) Wild-type mice received 1×10^6^ MCA-205 sarcoma tumor cells (n = 8/group). Tumor-bearing mice were treated with anti-OX40 or rat IgG Ab (days 10, 14; i.p.) along with IL-2 cytokine/mAb complexes (days 10–13; i.p.) and the extent of **A**) tumor growth and **B**) survival of tumor-bearing mice were assessed. Data are representative of one out of 2 independent experiments. **C**) MCA-205 tumor-bearing mice (as in (**A**)) received no treatment (n = 9), anti-CD4 (n = 6), anti-CD8 (n = 6), or anti-CD4+anti-CD8 (n = 3) (200 mcg/dose; i.p.) 9, 17, and 24 days post-tumor implantation. Mice were then treated with anti-OX40 (days 10, 14) and IL-2c (days 10–13) and the extent of survival of tumor-bearing mice was assessed.

**Figure 7:**
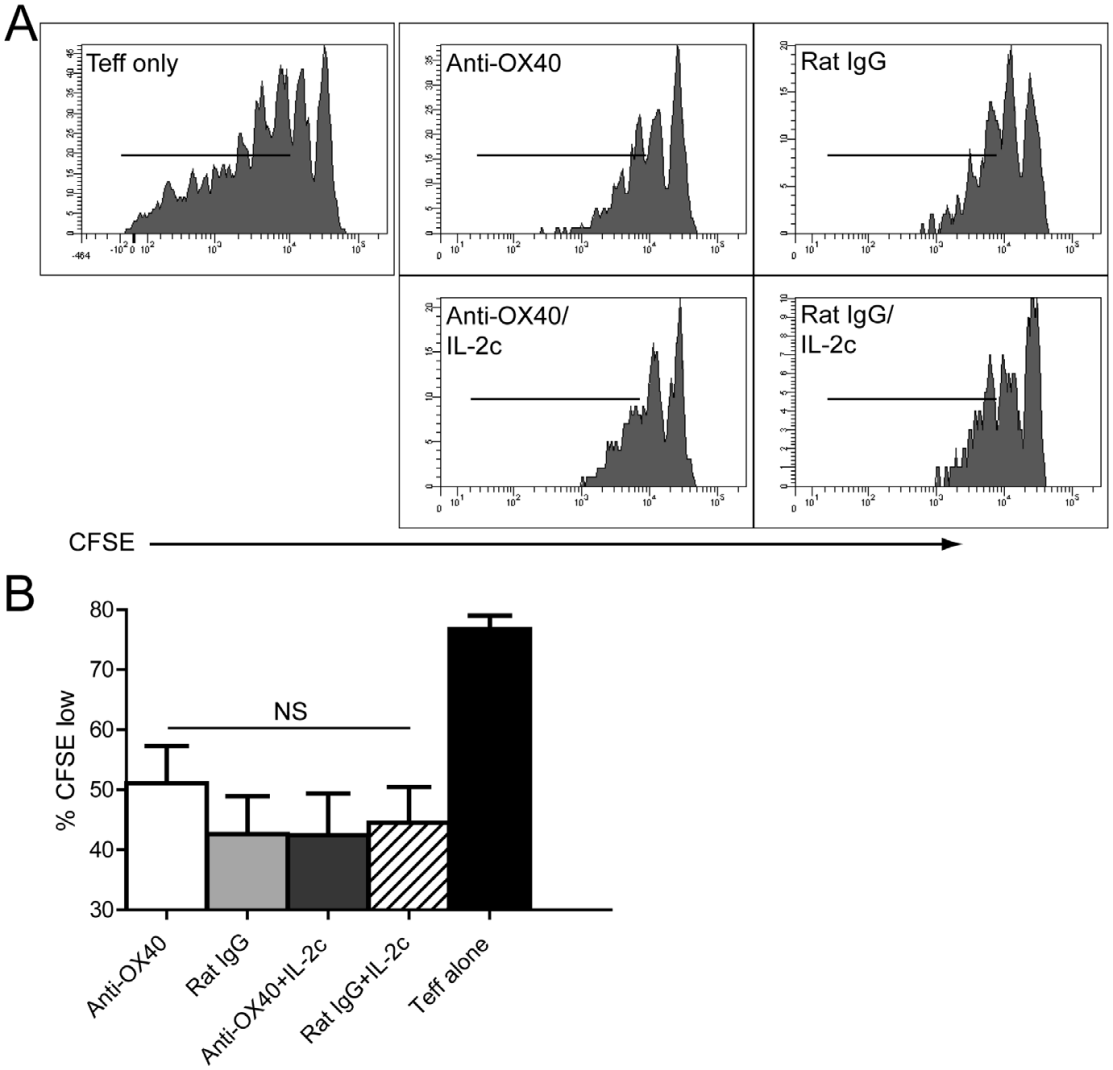
Treg functional assay. Wild-type mice received 1×10^6^ MCA-205 sarcoma tumor cells (n = 2–3/group). Tumor-bearing mice were treated with anti-OX40 or rat IgG Ab (days 10, 14; i.p.) along with IL-2 cytokine/mAb complexes (days 10–13; i.p.). On day 21, Treg were isolated from the spleens of tumor-bearing hosts and co-cultured with naïve CFSE-labeled responder CD8 T cells. Cells were harvested 96 hours later and the extent of CFSE dilution and in the CD8 responder cells was determined by flow cytometry. Graphs depict the results from A) individual mice or B) the mean+/−SD from n = 2–3/group.

Next, we tested the extent to which combined anti-OX40/IL-2 therapy would affect tumor growth and boost tumor immunotherapy. MCA-205 sarcoma tumor cells were implanted into wild-type mice and then 10 days later, mice were treated with anti-OX40 or control Ab and IL-2c. Importantly, tumor immunotherapy with combined anti-OX40/IL-2c significantly boosted tumor regression and survival compared to either treatment alone (Fig. 6A and 6B, respectively). To determine the on-target effects of dual anti-OX40/IL-2c therapy, CD4 and/or CD8 T cells were depleted from cohorts of tumor-bearing mice prior to providing anti-OX40/IL-2c therapy. Depletion of either CD4 or CD8 T cell subsets prior to anti-OX40/IL-2c therapy abrogated the anti-tumor efficacy of the treatment (Fig. 6C). Additional studies showed that combined anti-OX40/IL-2c therapy did not affect the accumulation or suppressive activity of CD4^+^CD25^+^ regulatory T cells (Fig. 7, Figure S2) demonstrating that effector CD4 and CD8 T cells are required for promoting tumor regression and enhanced long-term survival following dual anti-OX40/IL-2c immunotherapy.

### Dual anti-OX40/IL-2c therapy reverses CD8 T cell anergy and increases the survival of mice with long-term well-established tumors

**Figure 8:**
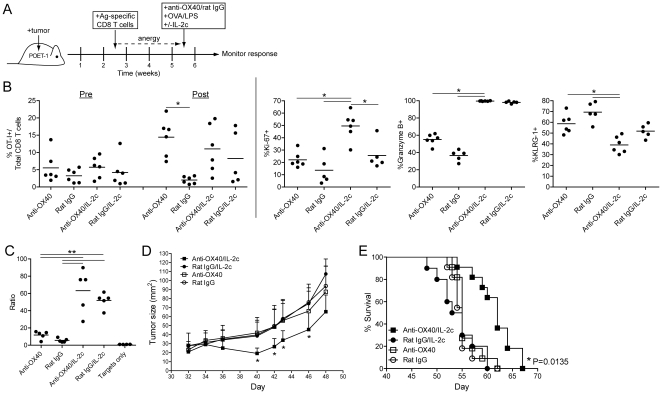
Dual anti-OX40/IL-2c therapy reverses CD8 T cell anergy and increases the survival of mice with long-term well-established tumors. **A**) Tumor model. 2.5×10^6^ TRAMP-C1-mOVA tumor cells were injected into POET-1 mice. Twenty days later, mice (∼50 mm^2^ tumors) received 5×10^5^ naïve OT-I T cells. Seventeen days after T cell adoptive transfer (37 days post-tumor inoculation), the anergic donor OT-I T cells were re-stimulated with anti-OX40 or control (rat IgG) Ab (d37-38), 500 mcg OVA (d37), 10 mcg LPS (d37), +/− IL-2 cytokine/mAb complexes (d37-44). **B**) Seven days after the initial dose of Ag/anti-OX40 the extent of donor CD8 T cell expansion (% OT-I of total CD8 T cells; pre- vs. post-treatment), Ki-67 (proliferation), granzyme B, and KLRG-1 expression on the donor OT-I T cells were determined. **C**) Tumor-bearing mice were treated as in (**B**) and then seven days later OVA peptide-pulsed (CFSE^high^) and control HA peptide-pulsed (CFSE^low^) target cells were mixed at a 1∶1 ratio and injected into recipient mice. Four hours later, spleens were harvested and the ratio of % CFSE^low^/% CFSE^high^ target cells from individual mice (n = 5/group) was determined. **D, E**) The extent of tumor growth (mean+/−SD; n = 5/group) and **D**) survival (n = 11/group) of tumor-bearing mice were assessed. Data are representative of one out of 2 to 3 independent experiments or **E**) the cumulative survival from 2 independent experiments. *P<0.05, **P<0.01.

Since tumor-induced T cell anergy is an important barrier that limits the generation of potent anti-tumor immunity [29], we sought to determine whether OX40 ligation in the presence of TCR/IL-2c signaling would restore the function of anergic CD8 T cells in tumor-bearing hosts. TRAMP-C1-mOVA expressing (TC1-mOVA) [16] prostate tumor cells were implanted in male POET-1 transgenic mice, in which prostate-specific expression of membrane-bound OVA (mOVA) is driven in an androgen-dependent manner [30]. This model allowed us to track the response of Ag-specific CD8 T cells against a surrogate tumor-associated Ag (see model; Fig. 8A). Twenty days later, donor OT-I T cells were adoptively transferred into the tumor-bearing hosts. Previous studies from our laboratory have shown that these tumor-reactive donor CD8 T cells become anergized *in vivo* [31]. Seventeen days after adoptive transfer (37 days post-tumor implantation), the tumor-bearing hosts were treated with anti-OX40 or rat IgG along with IL-2c. In addition, mice were given Ag/TLR ligand (LPS) to provide a source of TCR stimulation. The donor CD8 T cell response was determined 7 days later (timeline depicted in Fig. 8A).

The donor cells were rendered anergic as stimulation with Ag alone (rat IgG only) did not induce a proliferative response *in vivo* (Fig. 8B; compare pre- versus post-treatment; left panel) [31], while treatment with anti-OX40 and/or IL-2c was sufficient to promote expansion of the donor CD8 T cells (Fig. 8B; pre- versus post-treatment). Dual anti-OX40/IL-2c therapy significantly increased the proliferative response (Ki-67) and differentiation (GrzB) of the donor cells as compared to controls (Fig. 8B). No significant changes were detected in Treg accumulation among the anti-OX40 and/or IL-2 treated groups (Figure S2). Interestingly, further analysis revealed that the majority of cells receiving dual anti-OX40/IL-2c therapy exhibited a unique phenotype characterized by limited expression of the killer cell lectin-like receptor G1 (KLRG1) (Fig. 8B), which is typically highly expressed on terminally differentiated T cells that exhibit poor long-term survival [32], [33]. Anti-OX40/IL-2c therapy also led to a statistically significant increase in cytolytic activity as compared to anti-OX40 or rat IgG-treated controls (Fig. 8C) and dual anti-OX40/IL-2c treated cells trended towards increased cytolytic activity as compared to IL-2c treatment alone (Fig. 8C).

Finally, we examined the extent to which dual anti-OX40/IL-2c therapy affected tumor regression in mice with long-term well-established tumors (>40 days post-tumor implantation). These data revealed that combined anti-OX40/IL-2c therapy significantly enhanced tumor regression at several time points post-treatment (Fig. 8D) and also enhanced the survival of the tumor-bearing mice (Fig. 8E). Notably, this reflected a unique property of anti-OX40/IL-2 immunotherapy as treatment with anti-OX40/IL-4c or anti-OX40/IL-15c did not affect tumor growth or survival (data not shown). Together, these studies demonstrate that combined anti-OX40/IL-2c therapy can boost tumor immunotherapy by restoring the function of anergic tumor-reactive CD8 T cells *in vivo*.

## Discussion

Although studies have begun to elucidate the molecular mechanisms by which OX40 ligation augments T cell function, the mechanisms regulating OX40 expression itself remain poorly understood. In this study, we demonstrate that the initial expression of OX40 is regulated in part through the strength of TCR engagement, although TCR ligation alone was not sufficient to drive robust expression of OX40 (Fig. 1). Some studies showed that OX40 ligation enhanced IL-2 production and IL-2Ralpha on activated T cells, suggesting that IL-2R signaling may initiate a positive feedback loop that drives additional OX40 expression [6], [15], [16], although other work demonstrated that OX40 was expressed in a CD25-independent manner [10]. Thus, the precise role of IL-2R signaling in regulating OX40 remained unclear.

We sought to investigate the role of IL-2/IL-2R signaling in regulating OX40 expression and found that IL-2Ralpha-deficient T cells exhibited a marked defect in their ability to up-regulate OX40 following TCR ligation (Fig. 1C). Subsequent studies revealed that TCR ligation in the presence of IL-2 was sufficient to promote robust expression of OX40 on both murine and human CD4 and CD8 T cells (Fig. 2), suggesting that the IL-2-mediated enhancement of OX40 expression is likely part of a conserved mechanism of regulating OX40. In contrast to the transient expression of OX40 on activated T cells, OX40 is constitutively expressed on murine T_reg_ [34]. Although the reasons for this difference remain unclear, it may be related to alternative mechanisms that maintain the constitutive expression of CD25 on T_reg_, which may allow for regulation of OX40 through an IL-2R-dependent mechanism. OX40 expression could also be maintained on T_reg_ via chronic TCR engagement of self-peptide-MHC class II complexes regardless of IL-2 signaling. Several studies have revealed that T_reg_ can be detected in MHC class II-deficient mice although it remains unclear whether these cells maintain expression of OX40 [35], [36]. Interestingly, recent work from Piconese *et al.* described a reverse link between OX40 and IL-2 in Treg in which OX40-deficient Treg exhibited reduced competitive fitness compared to WT Treg *in vivo* [37]. OX40^−/−^ Treg also showed reduced expression of pSTAT5 following IL-2 stimulation, which was associated with increased expression of SOCS1, which is a negative regulator of STAT5 phosphorylation [38]. It will be of interest to examine whether there is also a role for reverse (OX40→IL-2) signaling in regulating effector CD8 T cells following stimulation with IL-2 or whether this is a unique property of Treg.

Mechanistic studies revealed that IL-2 stimulation induced JAK3 phosphorylation, which in turn was required for optimal induction of OX40 (Fig. 3A, 3B). Additional investigation demonstrated a hierarchy in which IL-2 consistently drove the most robust expression of OX40, while IL-4, IL-7, and IL-21 were less efficient at inducing OX40 (Fig. 3D). In contrast, IL-9 and IL-15 did not up-regulate OX40 (Fig. 3D). It should be noted that a similar hierarchy of gc cytokine-mediated induction of OX40 was obtained following stimulation of TCR Tg OT-I T cells or endogenous polyclonal CD8 T cells with wild-type pOVA (Fig. 3) or anti-CD3 (Fig. 4B). The molecular basis for the discordant effects of IL-15 versus IL-2/IL-4/IL-7/IL-21 stimulation remain unclear since all of these cytokines utilize at least partially overlapping signal transduction pathways via JAK3-mediated phosphorylation of STAT3 and STAT5 (Fig. 3E) [22], [23], [39]. Some possibilities include the regulation by adapter proteins like Gab2, negative regulators of STATs such as SOCS proteins, epigenetic changes, as well as differential activation and/or binding of STAT5alpha versus STAT5beta isoforms to the OX40 promoter [40], [41], [42].

We are also exploring whether differences in the homo- versus hetero-dimerization of STAT3 and STAT5 or in the binding of dimeric versus tetrameric STAT5 proteins to the OX40 promoter could account for differences in STAT3 versus STAT5-dependent induction of OX40 (Fig. 4) [43], [44]. To this end, we have identified putative STAT3 and STAT5-binding sites in the OX40 promoter and are beginning to elucidate the transcriptional machinery regulating OX40 expression. It should be noted that Sp1/Sp3/YY1 transcription factors can regulate the basal OX40 promoter, however this work did not address the role of gc/STAT-mediated signaling in driving OX40 expression [45].

Given the ability of IL-2 signaling to regulate OX40 expression *in vitro* and *in vivo*, we hypothesized that treatment with an agonist anti-OX40 mAb in conjunction with IL-2 would augment tumor immunotherapy. Indeed this was the case as combined anti-OX40/IL-2c therapy significantly enhanced tumor regression (Fig. 6A) and enhanced the survival of tumor-bearing hosts (Fig. 6B). The efficacy of dual anti-OX40/IL-2c therapy required the presence of effector CD4 and CD8 T cells in the tumor-bearing host as depletion of either or both subsets abrogated its effects (Fig. 6C) [16], [46]. Since anti-OX40 and IL-2 has been shown to modulate Treg function [47], [48], we sought to investigate the effects of dual anti-OX40/IL-2 therapy on Tregs in tumor-bearing hosts. Interestingly, we did not detect any change in Treg accumulation, suppressor function, or FoxP3 expression (% or MFI) following dual therapy (Fig. 7, Figure S2; and data not shown). A recent study provided some insight into these apparently discordant results by demonstrating that anti-OX40 therapy could turn off Treg-mediated suppression of CD4, but not CD8 T cell proliferation [49]. Given the critical role for effector CD8 T cells in promoting tumor rejection following dual anti-OX40/IL-2c therapy (Fig. 6C), the lack of change in Treg function suggests that alteration of Treg suppression is not the major mechanism by which dual therapy promotes tumor rejection. It should also be noted that the effects of IL-2c on Treg depend upon the specific clone used to generate the cytokine/anti-cytokine mAb complexes. Specifically, the JES6 clone of anti-IL-2 mAb enhanced Treg function since it preferentially binds to CD25 on T cells [26], [50], while use of the S4B6 clone (as used in this study) boosts primarily effector and memory T cells due to binding of CD122 (IL-2Rbeta) on T cells [26], [27], [51], [52].

Mechanistic studies revealed that dual anti-OX40/IL-2c therapy significantly increased the proliferation (Ki-67) and differentiation (granzyme B) of anergic tumor-associated Ag-specific CD8 T cells, while reducing their expression of the senescence-associated molecule KLRG1 (Fig. 8B). Although dual anti-OX40/IL-2c therapy and IL-2c treatment alone were both associated with increased cytolytic activity by the anergic CD8 T cells (Fig. 8C), only dual therapy led to increased anti-tumor activity in vivo as shown by increased tumor regression and survival of mice harboring long-term well established (>5 wks) tumors (Figs. 8D, 8E, respectively).

In summary, these data reveal that expression of the OX40 co-stimulatory molecule is controlled by a combined TCR/gc cytokine-dependent mechanism and that dual anti-OX40/IL-2 therapy was able to greatly augment tumor immunotherapy. Given the clinical development and availability of OX40 agonists and IL-2, it will be of great interest to translate these findings into the clinic and evaluate the therapeutic efficacy of combined anti-OX40/IL-2 therapy for the treatment of patients with cancer.

## Supporting Information

Figure S1 **Proliferation of wild-type versus CD25-/- CD8 T cells.** Naïve polyclonal wild-type or CD25^−/−^ CD8 T cells (3×10^5^/well) were CFSE-labeled and then stimulated with anti-CD3 and anti-CD28 (1 and 5 mcg/ml, respectively). One to three days later, CD8 T cells were harvested and the extent of proliferation (CFSE-dilution), **A**) CD25, and **B**) OX40 expression were determined. Data are representative of one out of two independent experiments with similar results.
(TIF)

Figure S2 **Effects of dual anti-OX40/IL-2c therapy on the accumulation of Treg.** **A**) 2.5×10^6^ TRAMP-C1-mOVA tumor cells were injected into POET-1 mice. Twenty days later, tumor-bearing mice received 5×10^5^ naïve OT-I T cells. Seventeen days after T cell adoptive transfer, the donor OT-I T cells were re-stimulated with anti-OX40 or control Ab, OVA/LPS, and IL-2 cytokine/mAb complexes (as in Fig. 8). **A**) The percentage of FoxP3^+^/CD4^+^ T cells in the peripheral blood (pre- and post-therapy) was determined by flow cytometry. **B**) When tumors progressed to >150 mm^2^, the tumor-bearing mice were euthanized and the percentage of FoxP3^+^ CD3^+^CD4^+^ T cells in the lymph nodes and tumor were determined by flow cytometry. Data represent the mean+/−SD from individual mice pooled from 2 independent experiments (n = 5–10). *P<0.01.
(TIF)
