# Supplementary figures and images for: Dual Anti-OX40/IL-2 Therapy Augments Tumor Immunotherapy via IL-2R-Mediated Regulation of OX40 Expression
Source: PLoS One. 2012 Apr 4;7(4):e34467. doi: 10.1371/journal.pone.0034467 (PMC3319580; doi:10.1371/journal.pone.0034467)

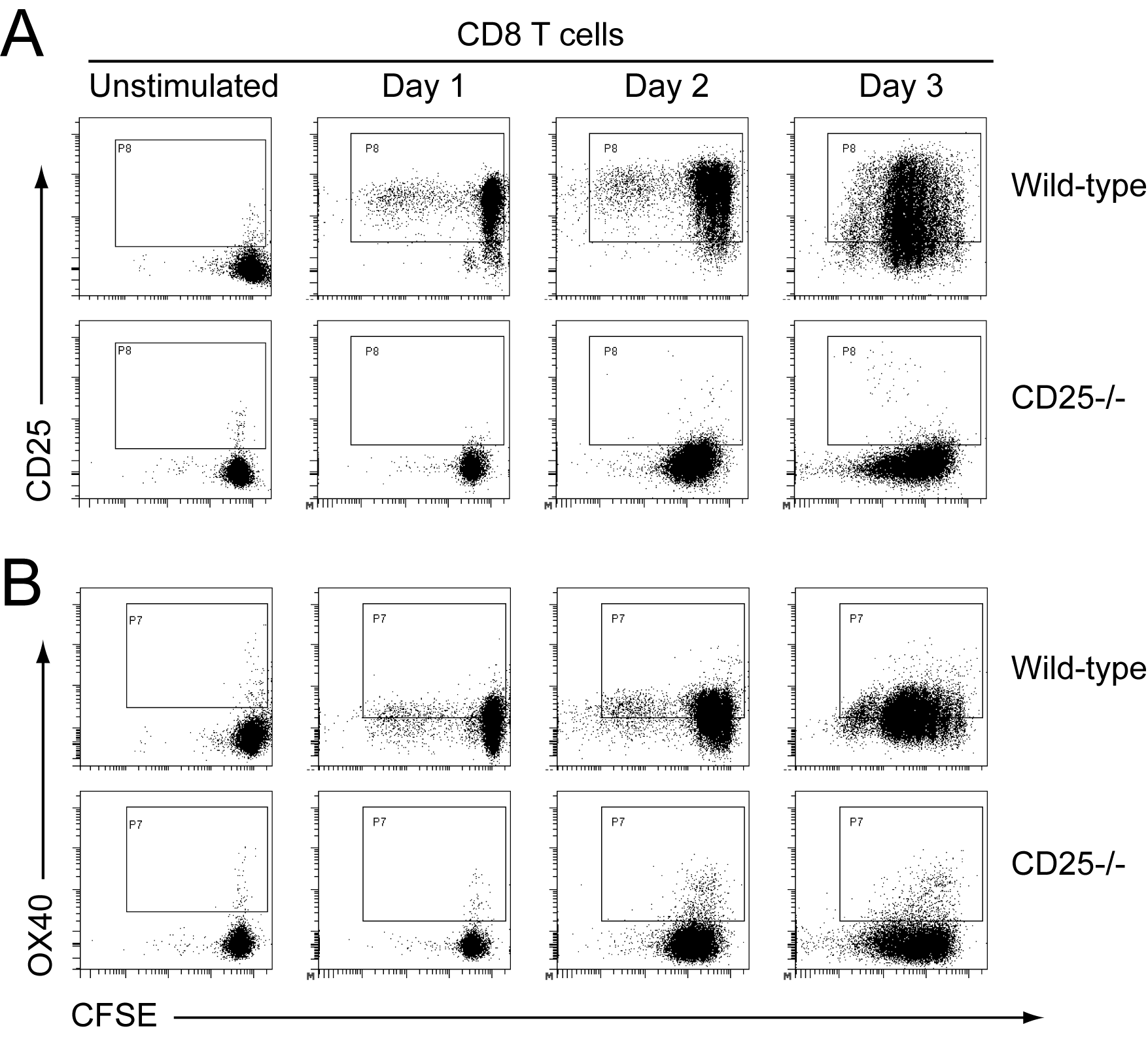

Supplement: Figure S1 — Proliferation of wild-type versus CD25-/- CD8 T cells. Naïve polyclonal wild-type or CD25−/− CD8 T cells (3×10^5^/well) were CFSE-labeled and then stimulated with anti-CD3 and anti-CD28 (1 and 5 mcg/ml, respectively). One to three days later, CD8 T cells were harvested and the extent of proliferation (CFSE-dilution), A) CD25, and B) OX40 expression were determined. Data are representative of one out of two independent experiments with similar results. (TIF) [file pone.0034467.s001.tif]

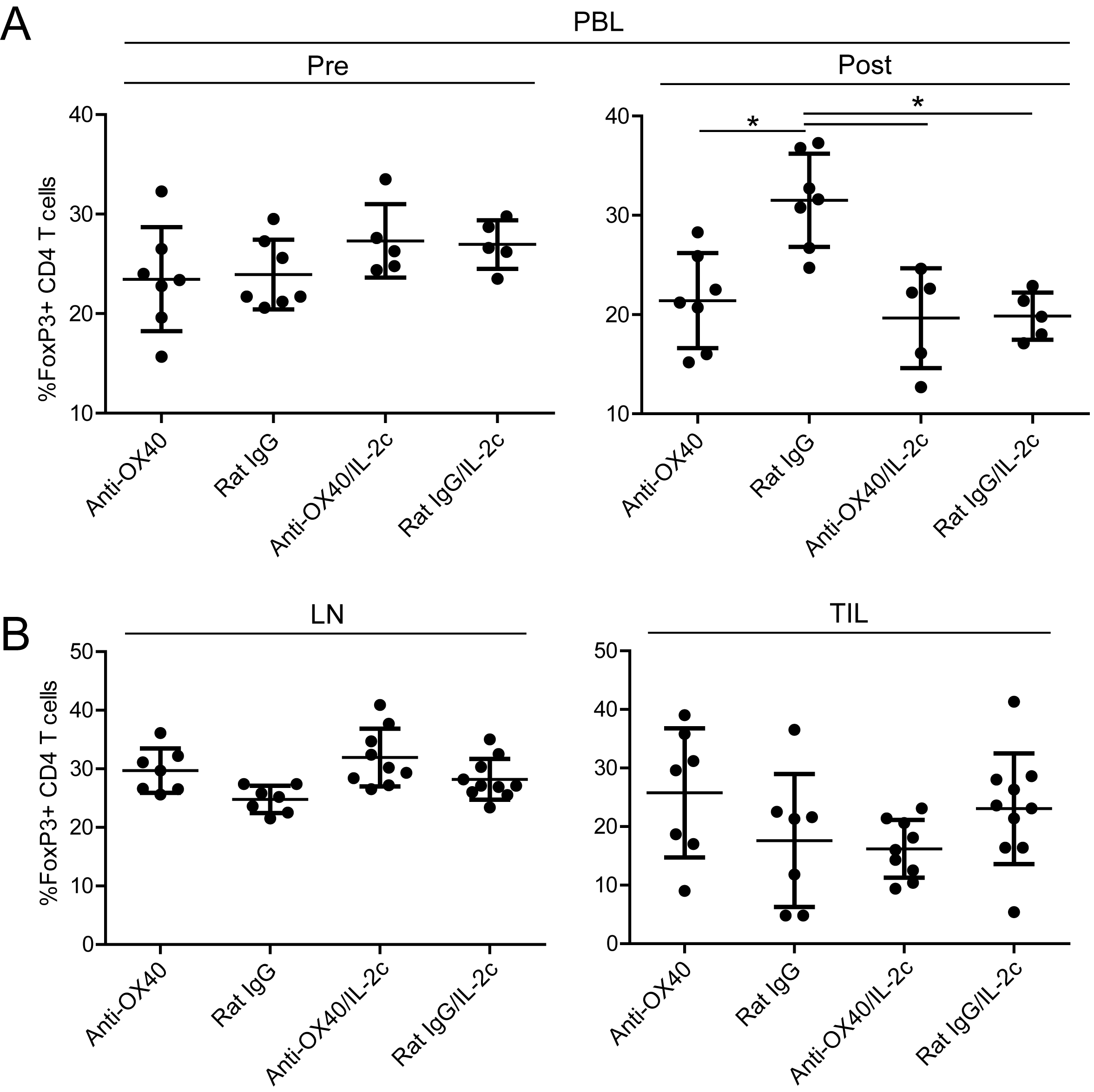

Supplement: Figure S2 — Effects of dual anti-OX40/IL-2c therapy on the accumulation of Treg. A) 2.5×10^6^ TRAMP-C1-mOVA tumor cells were injected into POET-1 mice. Twenty days later, tumor-bearing mice received 5×10^5^ naïve OT-I T cells. Seventeen days after T cell adoptive transfer, the donor OT-I T cells were re-stimulated with anti-OX40 or control Ab, OVA/LPS, and IL-2 cytokine/mAb complexes (as in Fig. 8). A) The percentage of FoxP3+/CD4+ T cells in the peripheral blood (pre- and post-therapy) was determined by flow cytometry. B) When tumors progressed to >150 mm2, the tumor-bearing mice were euthanized and the percentage of FoxP3+ CD3+CD4+ T cells in the lymph nodes and tumor were determined by flow cytometry. Data represent the mean+/−SD from individual mice pooled from 2 independent experiments (n = 5–10). *P<0.01. (TIF) [file pone.0034467.s002.tif]
